# PAK5-mediated phosphorylation and nuclear translocation of NF-κB-p65 promotes breast cancer cell proliferation in vitro and in vivo

**DOI:** 10.1186/s13046-017-0610-5

**Published:** 2017-10-17

**Authors:** Ying-Chun Zhang, Fu-Chun Huo, Lu-Lu Wei, Chan-Chan Gong, Yao-Jie Pan, Jie Mou, Dong-Sheng Pei

**Affiliations:** 1 Department of pathology, Xuzhou Medical University, Xuzhou, 221002 China; 2 Jiangsu Key Laboratory of Biological Cancer Therapy, Xuzhou Medical University, Xuzhou, 221002 China; 3 School of Pharmacy, Xuzhou Medical University, Xuzhou, 221002 China; 4 Department of Interventional Radiology, Jining No.1 People’s Hospital, Jining, Shandong Province China

**Keywords:** PAK5, p65, Cyclin D1, Cell proliferation

## Abstract

**Background:**

Abnormal proliferation is significantly associated with the promotion of malignant tumor. Growing evidence suggest that the signal pathways of p21^cdc42/rac1^-activated kinase 5 (PAK5) have been found in various tumor progression, however, the role of PAK5 in breast cancer remains largely unclear.

**Methods:**

We evaluated PAK5 and p65 staining in breast cancer tissues (BCTs) and paired non-cancerous tissues (NTs) using tissue microarray (TMA) technology. The functions of PAK5 were studied in vitro and in vivo. Cell Counting Kit-8 (CCK-8) and flow cytometry were performed to determine proliferation of breast cancer cells. Phosphorylation assay and co-immunoprecipitation (co-IP) were employed to identify the regulation mechanism of p65 by PAK5. The activation of Cyclin D1 promoter was measured with luciferase reporter assay. Xenograft models in nude mice were established to explore the roles of PAK5 in breast cancer growth.

**Results:**

In this study, we show that PAK5 is highly expressed in breast cancer tissues and the increased PAK5 is significantly associated with breast cancer progression. Overexpression of PAK5 promotes the proliferation and cell-cycle progression by increasing the expression of Cyclin D1 in vitro and in vivo*.* Mechanistic studies demonstrated that PAK5 can promote the phosphorylation and the nuclear translocation of p65 subunit of nuclear factor-kappaB (NF-κB). Furthermore, p65 can directly bind to the promoter of Cyclin D1 and mediate an increase in its protein expression.

**Conclusions:**

Taken together, our findings suggest that PAK5 may serve as a potential prognosis marker and therapeutic target for human breast cancer.

## Background

Breast cancer is currently the most frequently diagnosed cancer in female population, being also the second leading cause of cancer death among women worldwide [1]. Its survival rate descends from 90% for localized to 20% for metastatic disease [2]. Although early diagnosis and improved treatments have increased the survival of these patients, some breast cancer patients respond poorly to conventional treatments, leading to tumor recurrence and disease progression. Therefore, there is urgent need for us to unravel the molecular mechanisms underlying breast carcinogenesis and cancer progression, and thereby find appropriate biomarkers for targeted therapy.

P21^cdc42/rac1^-activated kinase 5 (PAK5) was first cloned and characterized in 2002 as a brain-specific kinase [3]. PAK5 gene is encoded by 12 exons in the *Homo sapiens* 20p12 chromosomal locus and encodes an 80 kDa protein. PAK5, being one of the members of PAK II subfamily of PAKs, localizes on mitochondria and the nucleus. Compared with other PAKs, PAK5 is the last identified and the least understood member [4, 5]. Major signal pathways of PAK5 have been found in tumor progression, which include the regulation of cytoskeleton changes, anti-apoptosis and proliferation in tumor cells [5, 6]. Knockdown of PAK5 inhibited human breast cancer cell proliferation by inducing cell cycle arrest in G0/G1 phase, which is generally in concordance with the downregulation of Cyclin D1 [7]. The underlying mechanisms of PAK5 on breast cancer cell proliferation, however, still remains to be fully elucidated. Thus, it is of great clinical value to further understand the molecular mechanisms involved in breast cancer and to find valuable diagnostic markers and novel therapeutic targets.

Nuclear factor-kappaB (NF-κB) is important for genes involved in cell survival, adhesion, differentiation, and proliferation. The mammalian NF-κB family is composed of five protein members: NF-κB1 (p50 and its precursor p105), NF-κB2 (p52 and its precursor p100), RelA (p65), RelB, and c-Rel [8]. They all share a Rel homology domain (RHD) essential for dimerization as well as binding to specific DNA sequences known as κB site which situates in promoter and enhancer regions of diverse genes [9]. In resting cells, inactive NF-κB is sequestered mainly in the cytoplasm in a complex with its inhibitory protein known as the inhibitor of κB (IκB). Phosphorylation and degradation of IκB in cytoplasm is required for the activation and nuclear translocation of NF-κB. Upon activation, NF-κB complex then translocates into the nucleus to activate target gene expression. Accumulating evidence has demonstrated that constitutive NF-κB activation has been noted in 95% of all cancers [10–12].

In the current study, we evaluated PAK5 and p65 staining in breast cancer tissues (BCTs) and paired non-cancerous tissues (NTs) using tissue microarray (TMA) technology and analyzed the correlation between PAK5 as well as p65 expression and clinicopathologic features. We characterized that PAK5 could promote the phosphorylation and the nuclear translocation of p65 subunit of nuclear factor-kappaB, and demonstrated that p65 could directly bind to the promoter of Cyclin D1. Furthermore, xenograft models in nude mice were established to explore the roles of PAK5 in breast cancer growth. Significantly, we showed that overexpression of PAK5 could promote the proliferation and cell-cycle progression by increasing the expression of Cyclin D1 in vitro and in vivo*.* Our data provide important insight into the PAK5-p65 signals in regulating Cyclin D1 to promote breast cancer growth.

## Methods

### Patients and specimens

Tissue specimens consisted of 129 breast cancer tissues (BCTs) and 46 adjacent non-cancerous tissues (NTs) were utilized in this study. All patients who were primary breast cancer underwent surgical operation without prior treatment at Affliated Hospital of Xuzhou Medical University between 2004 and 2008 and were followed up for at least 2 months, and up to 119 months or until mortality. Patients have complete clinical information including age, pathological tumor size (pT), pathological lymph node metastasis (pN), tumor node metastasis (TNM) stage and survival, which were pathologically confirmed. This study was performed under a protocol approved by the Review Board of the Affliated Hospital of Xuzhou Medical University, and all examinations were performed after obtaining written informed consents.

### Immunohistochemistry

Streptavidin-peroxidase (Sp) method was used in immunohistochemistry assay following a standard Sp Kit (Zhongshan biotech, Beijing, China). Tissue microarray (TMA) slides were dewaxed at 60 °C for 2 h followed by two 10 min washes with xylene and then rehydrated with different concentrations of ethanol including 100%, 95%, 85%, 75%, 50% in sequence and finally with distilled water for 5 min each. To retrieve antigen, the slides were put into 10 mM citrate buffer (pH 6.0) when the buffer was heated to 95 °C, and were kept for 30 min. Then, the endogenous peroxidase activity was inhibited by 3% hydrogen peroxide for 30 min. After blocking with 5% normal goat serum for 30 min, the slides were incubated with polyclonal rabbit anti-PAK5 antibody (1:100 dilution; Abcam, Shanghai, China) or p65 (1:50 dilution; Santa Cruz, Shanghai, China) overnight at 4 °C. The sections were then incubated for 1 h with a biotinlabeled secondary antibody, followed by avidin-peroxidase reagent and 3, 3′-diaminobenzidine (DAB; Zhongshan biotech) substrate. After hematoxylin counterstain and Dehydration, the sections were sealed with cover slips.

### Evaluation of immunostaining

For the TMA staining evaluation, the immunoreactivity was assessed blindly by two independent observers using light microscopy (Olympus BX-51 light microscope), and the image was collected by Camedia Master C-3040 digital camera. Positive PAK5 immunostaining is defined as cytoplasmic with or without nuclear staining and graded according to both the intensity and percentage of cells with positive staining. The PAK5 staining intensity was scored 0–3 (0 = negative; 1 = weak; 2 = moderate; 3 = strong). The percentage of PAK5-positive stained cells was also scored into four categories: 1 (0–25%), 2 (26–50%), 3 (51–75%) and 4 (76–100%). The level of PAK5 staining was evaluated by the immunoreactive score (IRS), which is calculated by multiplying the scores of staining intensity and the percentage of positive cells. Based on the IRS, staining pattern was defined as negative (IRS: 0), weak (IRS: 1–3), moderate (IRS: 4–6), and strong (IRS: 8–12).

### Cell culture

Human breast cancer cell lines MDA-MB-231 and BT549 were purchased from the Shanghai Institute of Biochemistry and Cell Biology, Chinese Academy of Sciences (Shanghai, China). MDA-MB-231 cells were cultured in Leibovitz’s L-15 medium supplemented with 10% fetal bovine serum (Invitrogen, Shanghai, China); BT549 cells were cultured in RPMI-1640 (GIBCO, Shanghai, China) supplemented with 10% fetal calf serum. Cells were in a 37 °C humidified incubator with 95% air, 5% CO_2_.

### Transfection

Non-specific control siRNA and PAK5 siRNA were purchased from Integrated Biotech Solutions (Shanghai, China). The sequences of siRNAs are as follows: PAK5, 5′-CAAAGTCTTCGTACCTGAATC-3′ [13]. The cells were transfected with siRNA using siLentFect Lipid Reagent (Bio-Rad, Hercules, CA, USA). The pcDNA3.1-Myc-control, pcDNA3.1-p65 plasmids and pcDNA3.1-Myc-PAK5 expression plasmids were purchased from Guangzhou FulenGen Co. BT549 and MDA-MB-231 cells were grown to 90% confluence before being transiently transfected with PAK5 plasmids using X-tremeGENE HP DNA Transfection Reagent (Roche, Indianapolis, IN, USA). After transfection with PAK5 siRNA or PAK5 overexpression plasmid, cells were harvested for subsequent experiments.

### Cell proliferation assay

Cell proliferation was assayed with the Cell Counting Kit-8 (CCK-8) kit (Vicmed, Xuzhou, China). After transfection, BT549 and MDA-MB-231 cells (4 × 10^3^) were cultured in 96-well microplate (Corning Incorporated, NewYork, USA) in triplicate. Then, CCK-8 reagent (10 μl) solution with 100 μl serum-free medium added to each well at 24, 48, 72, and 96 h, respectively, followed by incubation for 2 h at 37 °C. The absorbance at 450 nm was measured to calculate the cell viability by a multi-function enzyme-linked analyzer (Biotek Instruments, Winooski, VT, USA).

### Cell-cycle analysis by flow cytometry

BT549 and MDA-MB-231 cells were harvested and washed with cold PBS three times after transfected with control siRNA/PAK5 siRNA or pcDNA3.1-Myc-control/pcDNA3.1-Myc-PAK5 expression plasmids. Then, the cells were fixed with 70% cold ethanol at 4 °C overnight. Subsequently, the cells were centrifuged and resuspended in 500 μl DNA staining solution and incubated for 30 min at 37 °C in the dark, followed by the addition 10 μl propidium iodide. Samples were then analyzed using FACScan flow cytometer (BD Biosciences, San Jose, CA, USA). Data on cell cycle distribution were analyzed using ModFit LT 3.0 software.

### Nuclear cytoplasmic fractionation

The extraction and isolation of nuclear and cytoplasmic protein were performed according to the Nuclear and Cytoplasmic Protein Extraction Kit (Beyotime, Shanghai, China) and its manufacturer’s instructions.

### Antibodies, Western blot and co-immunoprecipitation (co-IP)

Antibodies against the following proteins were used: PAK5 (1:500; Abcam); p65 (1:500; Santa Cruz); β-actin (1:1000; Zhongshan biotech); Cyclin D1, c-Myc, Cyclin B1, Cyclin B3, Cyclin A1, Cyclin A, p21, p27 (1:200; Santa Cruz); Cyclin D3 (1:2000; Cell Signaling Technology, Beverly, MA, USA); Anti-rabbit HRP, Anti-Mouse HRP (1:10,000, Vicmed). After performing specific treatments, cells were washed two times with PBS and were harvested using RIPA lysis buffer (Beyotime). Cell lysates was pelleted by centrifugation at 15,000 *g* for 15 min at 4 °C, the supernatants were collected and the protein concentrations were determined using the bicinchoninic acid kit (BCA, Pierce, USA) according to the manufacturer’s instructions. Proteins were separated by electrophoresis on SDS-polyacrylamide gel electrophoresis and electro-transferred to nitrocellulose filter membrane. After being blocked for 2 h in 5% bovine serum albumin, membranes were incubated overnight at 4 °C with the following primary antibodies: anti-PAK5, anti-c-Myc, anti-p65, anti-Cyclin A, anti-Cyclin A1, anti-Cyclin D1, anti-Cyclin D3, anti-Cyclin B1, anti-Cyclin B3, anti-p21, anti-p27 and anti-β-actin. Membranes were then washed and were probed with HRP conjugated secondary antibodies. The signals were detected using Chemistar™ High-sig ECL Western Blot Substrate (Tanon, shanghai, China). For co-IP, MDA-MB-231 Cells were transfected with the pcDNA3.1-Myc-control plasmids, pcDNA3.1-Myc-PAK5 plasmids and pcDNA3.1-p65 plasmids. Cell lysates (1000 μg) contained a cocktail of protease/phosphatase inhibitors (Sigma Aldrich, MO, USA) were used for immunoprecipitation for 12–16 h with anti-p65 and Rabbit IgG (Beyotime). Then 20 μl Protein A/G beads (Santa Cruz) were added in cell lysates and incubated for 2 h at 4 °C. Beads were washed with lysis buffer three times. Immunoprecipitated proteins were subjected to analysis described above.

### Immunofluorescence and confocal microscopy

Briefly, MDA-BM-231 and BT549 cells transfected with pcDNA3.1-Myc-control/pcDNA3.1-Myc-PAK5 expression plasmids were cultured on glass coverslips in a 24-well plate. Cells were fixed in 4% paraformaldehyde for 20 min at room temperature, and then rinsed with PBS 15 min. After being permeabilized with 0.3% Triton for 15 min, cells were blocked with PBS containing 5% BSA for 30 min. Subsequently, Cells were incubated with the primary antibodies p65 (anti-mouse, 1:300; Santa Cruz) diluted in blocking buffer overnight at 4 °C, followed by visualization with Alexa Fluor 488-goat anti-mouse IgG secondary antibody (30 min) diluted 1:100 in blocking buffer. Nuclei were stained with 4′, 6-Diamidino-2-phenylindole (DAPI) for 10 min. Images were examined and recorded using immunofluorescence confocal laser scanning microscopy (Zeiss LSM 880).

### Phosphorylation assay

MDA-MB-231 cells were transfected with the pcDNA3.1-Myc-control and pcDNA3.1-Myc-PAK5 plasmids. Cell lysates contained a cocktail of protease/phosphatase inhibitors (Sigma Aldrich). Partial Cell lysates were treated with Alkaline Phosphatase (AP) (Thermo Scientific, Shanghai, China) and then incubated for about 12 h at 37 °C. Proteins were separated by electrophoresis on Mn^2+^–Phos-tagTM SDS-PAGE [14]. The following is the same as corresponding Western blot procedure described above.

### Luciferase reporter assay

Cells were split into 48-well plates, and each well was co-transfected with 200 ng luciferase vector pGL3-Basic as control, Cyclin D1 promoter −1000/−1, together with 800 ng pcDNA3.1 as control, pcDNA3.1-p65 plasmids. After 24 h, whole-cell lysate was collected for reporter detection by the Dual Luciferase Reporter System (Promega, Shanghai, China). Reactions were measured using an Orion Microplate Luminometer (Berthold Detection System). Transfections were performed in triplicate, and repeated three times to assure reproducibility.

### Establishment of tumor xenografts in nude mice

Twelve five-week-old female BALB/c nude mice were purchased from Beijing Huafukang Bioscience (Beijing, China) and maintained under a specific pathogen-free facility conditions. Animal experiments were in conformance with the Institutional Animal Care and Use Committee of Xuzhou Medical University. Vector control/PAK5-espressing MDA-MB-231 (1 × 10^6^) cells suspended in 150 μl liquid medium and were injected subcutaneously into the armpit of mice. Tumor volumes were assessed twice weekly. All mice were sacrificed after 35 days. Tumor samples then treated for immunohistochemical staining. Meanwhile, extraction proteins of the resected tumor samples were performed using Trizol Reagent (Thermo Fisher Scientific, US) for Western blot.

### Statistical analysis

Two-factor analysis of variance procedures and the Dunnett’s t-test were used to assess differences within treatment groups. For TMA, statistical analysis was performed with SPSS 16.0 software (SPSS, Inc., Chicago, IL, USA). The association between PAK5 or p65 staining and the clinicopathologic parameters of the breast cancer patients, including ages, pT status, pN status and TNM stage, was evaluated by two sided Fisher’s exact tests. Differences in IRS for PAK5 and p65 staining in primary BCTs and their adjacent NTs were assessed by the Wilcoxon test (grouped). The Kaplan-Meier method and log-rank test were used to evaluate the correlation between PAK5 expression and patient survival. The correlation analysis between PAK5 and p65 was evaluated by spearman test. Data represent mean ± SD. Differences were considered statistically significant when *P* < 0.05.

## Results

### The expression of PAK5 and p65 are both increased in human breast cancer

**Fig. 1:**
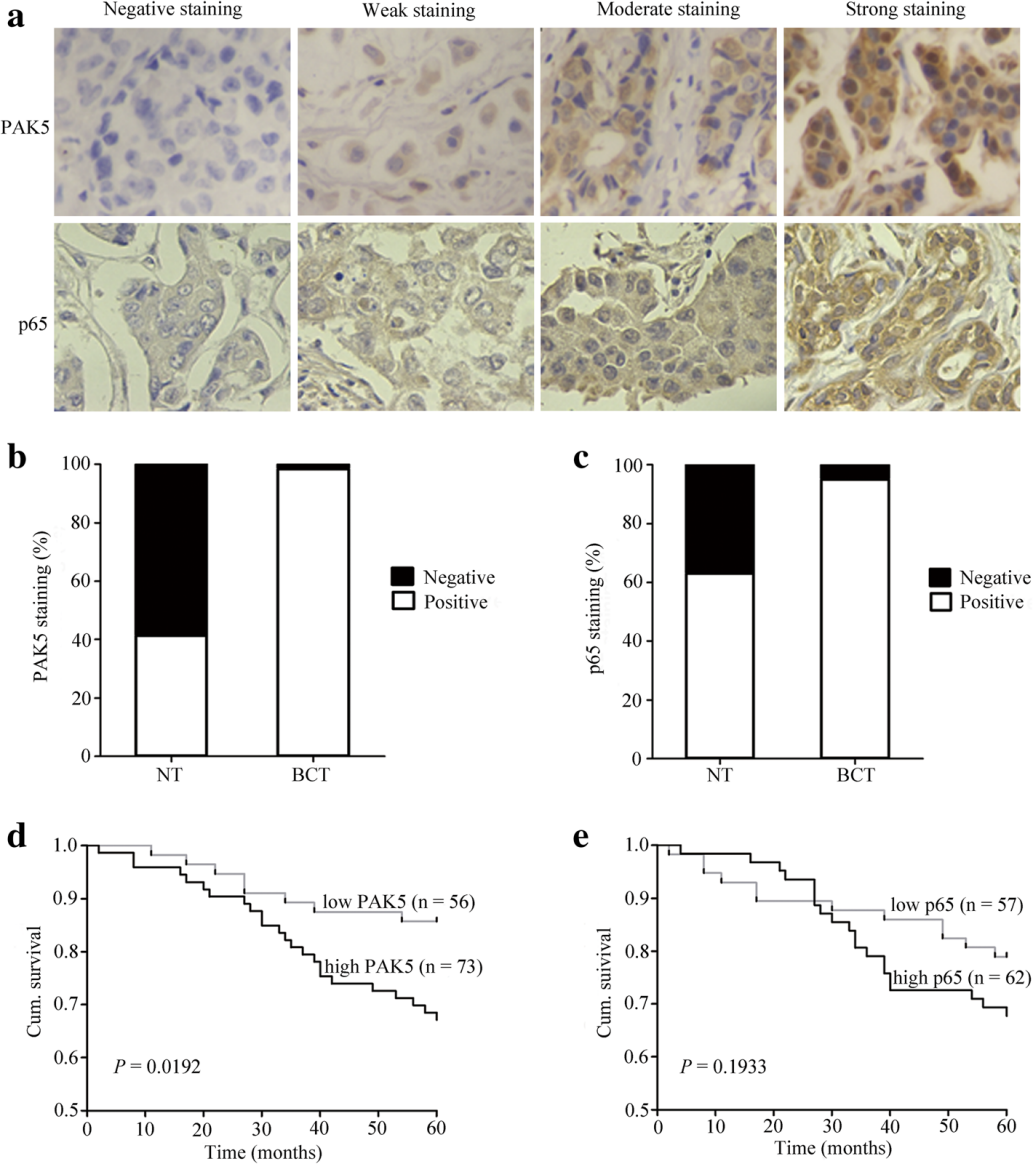
PAK5 correlates with a worse prognosis in breast cancer patients. **a** Representative images of PAK5 and p65 immunohistochemical staining in human breast cancer. Magnification × 400 for a. **b** PAK5 expression staining was higher in BCTs than in paired adjacent NTs. **c** p65 expression staining was higher in BCTs than in paired adjacent NTs. **d** High PAK5 expression correlated with a poorer 5-year overall cumulative survival for 129 breast cancer patients (*P =* 0.0192, log-rank test). **e** High p65 expression did not correlate with 5-year overall cumulative survival for 119 breast cancer patients (*P* = 0.1933, log-rank test)

To investigate PAK5 and p65 expression in breast cancer, immunohistochemistry was performed in TMA slides containing 129 BCTs and 46 paired adjacent NTs. The immunohistologic staining of PAK5 and p65 in Fig. 1a classified as negative, weak positive, moderate positive and strong positive. Samples with IRS 0–4 and IRS 6–12 were classified as low and high expression of PAK5 or p65. PAK5 positive expression staining was observed in 127 of 129 (98.4%) BCTs, and 19 of 46 (41.3%) adjacent NTs (Fig. 1b). p65 positive expression staining was observed in 110 of 119 (92.4%) BCTs, and 26 of 46 (56.5%) adjacent NTs (Fig. 1c). These results showed that PAK5 and p65 expression were both increased in human BCTs compared with paired adjacent NTs.

### The correlation of PAK5 and p65 expression with clinicopathologic parameters

The relationship between PAK5, p65 and various clinicopathologic features were investigated in TMA slides with 129 cases or 119 cases respectively. Of the 129 breast cancer patients analyzed, low and high PAK5 staining were 43.4% (56/129) and 56.6% (73/129), respectively. Of the 119 breast cancer patients analyzed, low and high p65 staining were 47.9% (57/119) and 52.1% (62/119), respectively (Table 1). As shown in Table 1, we found that both the increased PAK5 expression and the increased p65 expression was significantly correlated with some clinicopathological features, such as pT status (*P* = 0.000 for PAK5, *P* = 0.039 for p65), pN status (*P* = 0.011 for PAK5, *P* = 0.018 for p65), and TNM stage (*P* = 0.030 for PAK5, *P* = 0.018 for p65). However, we did not find a significant association of PAK5 or p65 expression levels with patient’s age.

**Table 1:** PAK5 and p65 staining and clinicopathologic characteristics of breast cancer patients

	PAK5 staining (*n* = 129)				p65 staining (*n* = 119)			
Variables	Low (%)	High (%)	Total	*P value*	Low (%)	High (%)	Total	*P* value
Age								
< 50 years	14 (38.9)	22 (61.1)	36	0.558	13 (40.6)	19 (59.4)	32	0.409
≥ 50 years	42 (45.2)	51 (54.8)	93		44 (50.6)	43 (49.4)	87	
pT status								
pT_0_-pT_1_	23 (69.7)	10 (30.3)	33	0.000	21 (63.6)	12 (36.4)	33	0.039
pT_2_- pT_4_	29 (30.9)	65 (69.1)	94		34 (40.5)	50 (59.5)	84	
pN status								
pN_0_	34 (50.7)	33 (49.3)	67	0.011	36 (59.0)	25 (41.0)	61	0.018
pN_1_- pN_3_	17 (27.9)	44 (72.1)	61		21 (36.8)	36 (63.2)	57	
TNM stage								
I~II	32 (50.8)	31 (49.2)	63	0.03	36 (59.0)	25 (41.0)	61	0.018
I~III	11 (32.4)	23 (67.6)	34		14 (43.8)	18 (56.2)	32	
III	5 (21.7)	18 (78.3)	23		4 (22.2)	14 (51.4)	18	

pT pathological tumour size, pN pathological lymph node metastasis, TNM Tumor node metastasis

### PAK5 serves as a potential prognostic indicator for breast cancer

To further study whether increased PAK5 staining in breast cancer patients correlates with a worse prognosis. Survival analysis was performed using 5-year overall survival by Kaplan-Meier survival analysis and log-rank test. Our data revealed that high PAK5 staining correlated with worse overall survival in breast cancer (*P* = 0.0192, Fig. 1d). The 5-year overall cumulative survival rate dropped from 85.7% in patients with low PAK5 expression to 67.1% in those with high PAK5 expression. However, we did not find a significant association of p65 expression level with overall survival in breast cancer (Fig. 1e).

We further investigate the relationship between PAK5 and p65 expression and results show that PAK5 and p65 are positively related to each other (*r* = 0.326, *P* < 0.05), which implies that the coordination between PAK5 and p65 expression may have a positive impact on breast cancer progression (Table 2).

**Table 2:** The correlation of PAK5 and p65 expression in human breast cancer

	PAK5		Spearman	
p65	Low	High	Rho	*P* value
Low	33	24	0.361	0.000
High	14	48		
Total	47	72		

### PAK5 promotes proliferation and cell-cycle progression by increasing the expression of Cyclin D

**Fig. 2:**
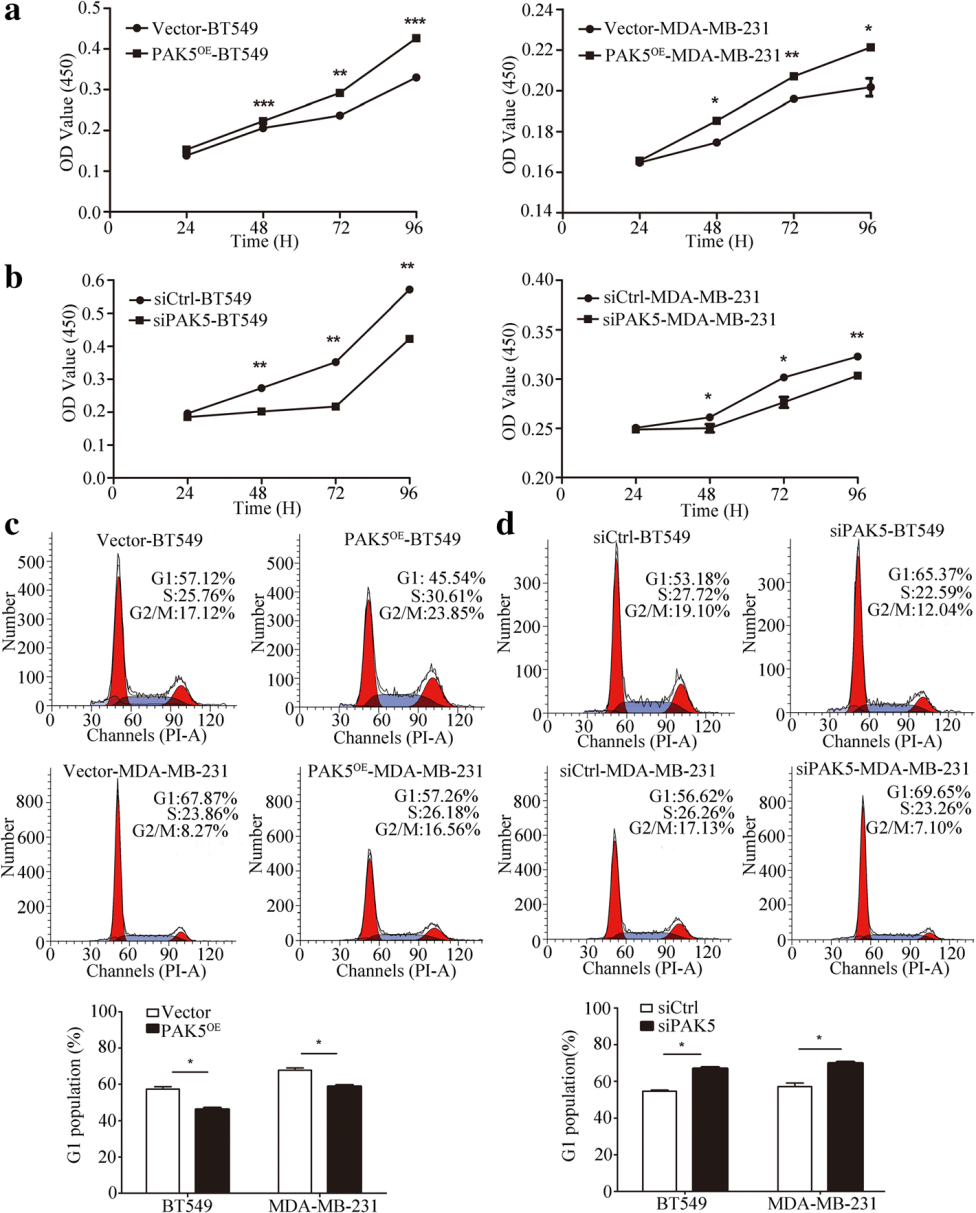
The effect of PAK5 on breast cancer cells proliferation. **a** CCK-8 cell proliferation assays after PAK5 overexpression in BT549 and MDA-MB-231 cells. **b** CCK-8 cell proliferation assay after PAK5 knockdown in BT549 and MDA-MB-231 cells. **c** Effects of PAK5 overexpression on the cell cycle of breast cancer cells. The percentage of G1 population cells was measured by flow cytometry after PAK5 overexpression in BT549 and MDA-MB-231 cells. **d** Effects of PAK5 silencing on cell cycle analysis of BT549 and MDA-MB-231 cells. The percentage of cells at G1 stage was calculated using ModFit LT 3.0 software. Data are presented as mean ± SD for three independent experiments. *, *P* < 0.05; **, *P* < 0.01; ***, *P* < 0.001

Because dysregulation of cell cycle and out of control of cell division lead to infinite proliferation of cancer cell to some extent, we examined the effects of PAK5 on proliferation and cell cycle of breast cancer cells. Our results showed that overexpression of PAK5 could significantly accelerate cellular growth in both MDA-MB-231 and BT549 cells (Fig. 2a). Inversely, knocking down endogenous PAK5 dramatically restrained proliferative ability in both breast cancer cell lines compared with the negative control (Fig. 2b). In the following experiment, we further investigated whether cell cycle of breast carcinoma cells changes resulting from regulation of PAK5 expression. As expected, the cell cycle analysis showed that overexpression of PAK5 decreased the percentage of cells in G1 phase (Fig. 2c); conversely, the proportion of cells in G1 phase increased upon PAK5 gene silencing (Fig. 2d). These results suggested that PAK5 could promote cell proliferation in breast cancer cells by speeding up G1-S transition.

**Fig. 3:**
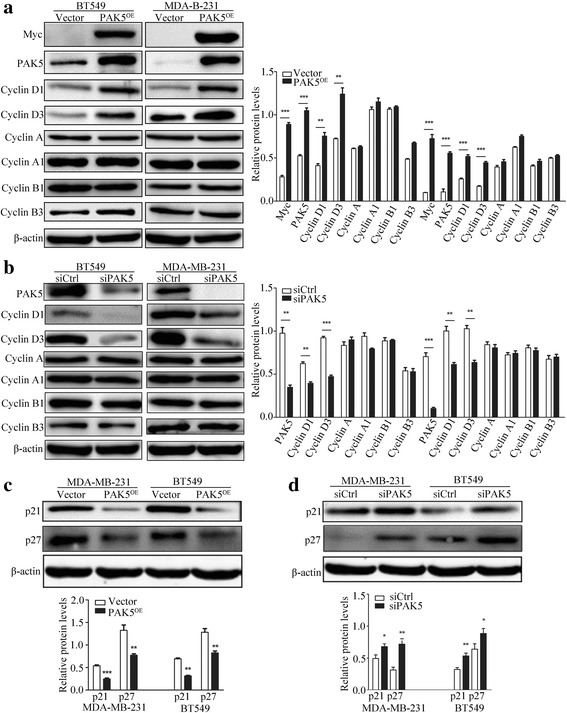
Impact of PAK5 on the expression of cell cycle regulatory proteins. **a**, **b** Western blot analysis of the PAK5 gene overexpression and silencing on Myc, PAK5, Cyclin D1, Cyclin D3, Cyclin A, Cyclin A1, Cyclin B1 and Cyclin B3 in BT549 and MDA-MB-231 cells. **c**, **d** Western blot analysis of the PAK5 gene overexpression and silencing on p21 and p27 in BT549 and MDA-MB-231 cells. Data are shown as mean ± SD for three independent experiments. *, *P* < 0.05; **, *P* < 0.01; ***, *P* < 0.001

Cyclin D proteins are the initial factors of cell cycle that coordinate G1 progression with their catalytic partners CDK4/6, and they represent components of cell cycle machinery [15]. To further elucidate molecular mechanisms of PAK5 regulating proliferation and cell cycle in breast carcinoma cells, we performed Western blot to detect the Cyclin D proteins levels in MDA-MB-231 and BT549 cells (Fig. 3a, b). Our results showed that PAK5 positively regulated cyclin D1 and cyclin D3 and negatively regulated p21 and p27 (Fig. 3c, d). However, PAK5 did not affect the protein levels of Cyclin A1, Cyclin A, Cyclin B1 and Cyclin B3. Taken together, PAK5 can augment the expression of Cyclin D1 and Cyclin D3 and inhibit the expression of p21 and p27, accelerating cell-cycle progression in breast cancer cells.

### PAK5 facilitates the nuclear translocation of the NF-κB-p65

**Fig. 4:**
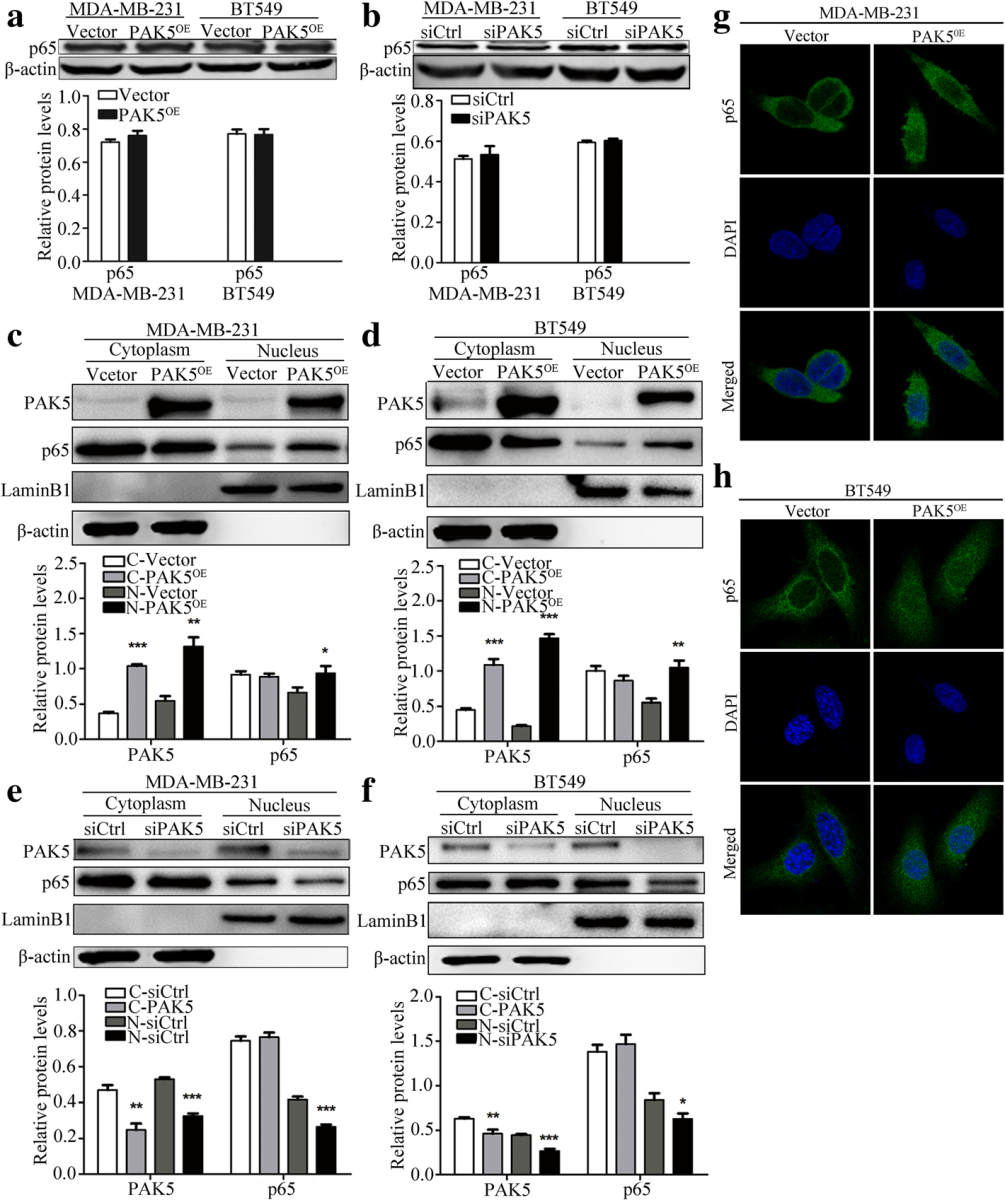
PAK5 activates the nuclear translocation of the p65. **a** Western blot of intracellular p65 from BT549 and MDA-MB-231 cells transfected with the pcDNA3.1-Myc-PAK5 plasmid or vector control. **b** Western blot of intracellular p65 from BC cells transfected with the PAK5 siRNA or control siRNA. **c**, **d** Western blot determined cellular distribution of p65 in BT549 and MDA-MB-231 cells transfected with the pcDNA3.1-Myc-PAK5 plasmid or vector control. **e**, **f** Western blot determined cellular distribution of p65 in BC cells transfected with PAK5 siRNA or control siRNA. **g**, **h** Immunofluorescent staining of MDA-MB-231 and BT549 cells analyzed by confocal microscopy (magnification, × 400). The antigenic sites of p65 were detected using Alexa Fluor 488 conjugated goat anti-mouse IgG (green). Data are shown as mean ± SD for three independent experiments. *, *P* < 0.05; **, *P* < 0.01; ***, *P* < 0.001

**Fig. 5:**
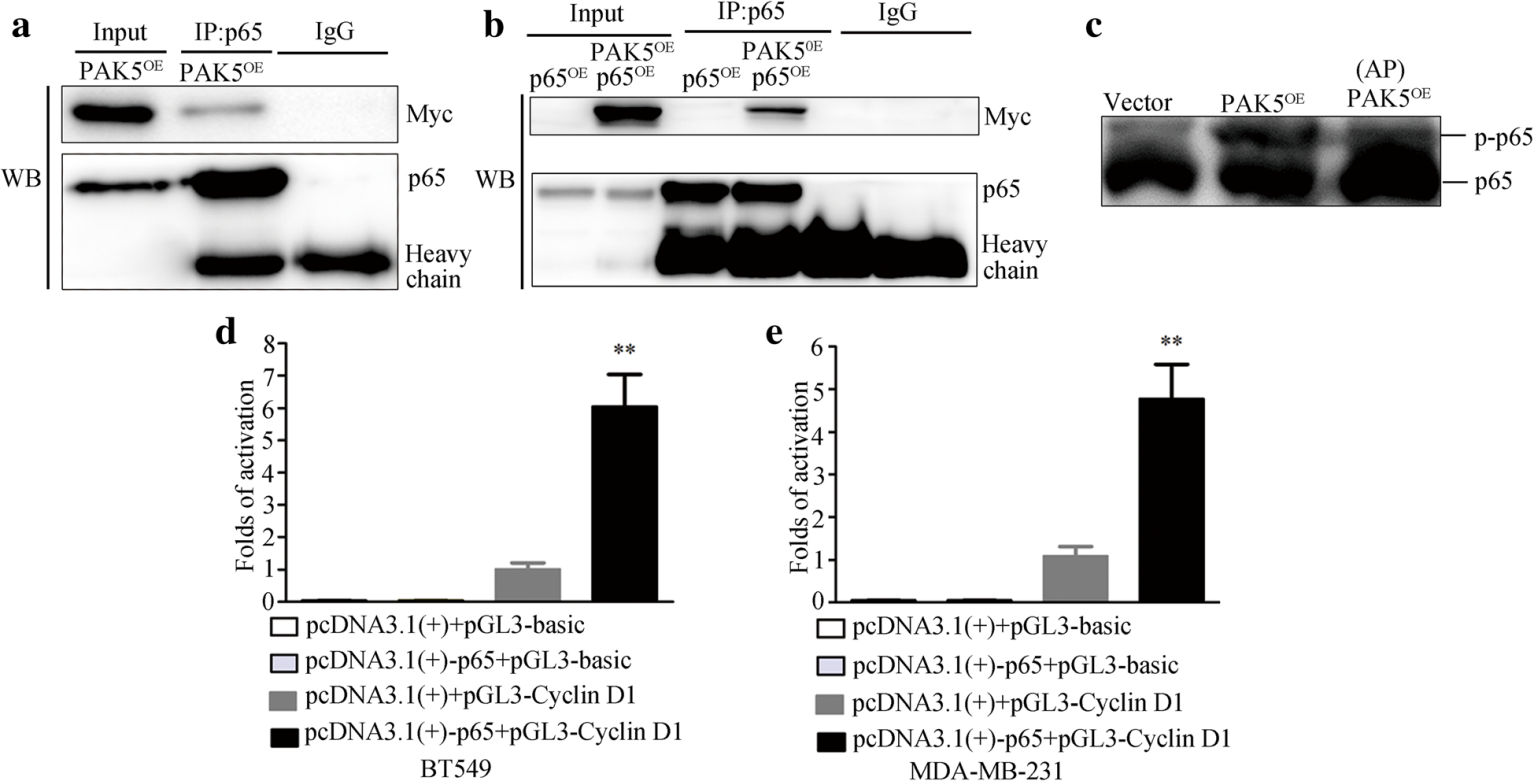
PAK5 interacts and phosphorylates p65. **a** MDA-MB-231 cells were transfected with pcDNA3.1-Myc-control plasmids and pcDNA3.1-Myc-PAK5 plasmids. Lysates were immunoprecipitated with anti-p65 antibody and immunoblotted with anti-Myc and anti-p65 antibody. **b** MDA-MB-231 cells were transfected with pcDNA3.1-Myc-control plasmids, pcDNA3.1-p65 plasmids and pcDNA3.1-Myc-PAK5 plasmids. Lysates were immunoprecipitated with anti-p65 antibody and immunoblotted with anti-Myc, and anti-p65 antibody. **c** MDA-MB-231 cells transfected with pcDNA3.1-Myc-control plasmids and pcDNA3.1-Myc-PAK5 plasmids. Then concentrated protein was used for WB. The top p-p65 represents the mobility shift of phosphorylated p65 proteins in SDS-PAGE with polyacrylamide-bound Mn^2+^-Phos-tag. The bottom p65 represents non-phosphorylated counterpart in SDS-PAGE with polyacrylamide-bound Mn^2+^-Phos-tag. **d**, **e** Luciferase reporter assay was carried out in BT549 and MDA-MB-231 cells. The relative luciferase activities of pcDNA3.1 + pGL3-basic, pcDNA3.1-p65 + pGL3-basic and pcDNA3.1-p65 + pGL3-Cyclin D1 groups normalized against pcDNA3.1 + pGL3-Cyclin D1 group. Data are shown as mean ± SD for three independent experiments. **, *P* < 0.01

Previous studies indicate that Cyclin D1 is an important downstream target of NF-κB that participates in the early phases of the cell cycle to regulate cell growth and plays a vital role in cancer initiation and progression [16–18]. In order to investigate the underlying mechanism of PAK5 in proliferation, we examined the expression of p65 in MDA-MB-231 and BT549 cells transfected with PAK5 or siPAK5. Unfortunately, Western blot analysis showed that the expression of total p65 was not obviously affected (Fig. 4a, b). Then, we carried out nuclear and cytoplasmic fractionation and observed the cellular distribution of p65 in MDA-MB-231 and BT549 cells by Western blot after PAK5 overexpression or knockdown. Our results showed that overexpression of PAK5 significantly increased the nuclear distribution of p65 (Fig. 4c, d). In contrast, silence of PAK5 markedly decreased the nuclear distribution of p65 (Fig. 4e, f). Consistently, results from immunofluorescence showed that positive signal (green) of p65 was increasingly detected in the nucleus after the overexpression of PAK5, whereas this positive signal was predominantly located in the cytoplasm with a small amount of weak nuclear staining in control groups, which fully demonstrated that upregulation of PAK5 expression enhanced p65 nuclear localization. According to these results, we assumed that there may be a potential and vague relationship between PAK5 and p65. To certify the underlying mechanisms, we performed phosphorylation and co-IP assay (Fig. 5a-c). The results showed that PAK5 could physically interact with p65, resulting in PAK5-mediated p65 phosphorylation. Collectively, these data indicated that PAK5 might increase the phosphorylation of p65 and then activate NF-κB-p65 translocation into nucleus, promoting Cyclin D1 expression.

### NF-κB-p65 targets Cyclin D1 promoter and actives Cyclin D1 transcription

p65 subunit of NF-κB is an important transcriptional activator of the cyclin D1 gene. Moreover, activated p65 can regulate Cyclin D1 by direct binding to multiple sites in the Cyclin D1 promoter at positions −858, −749, and −39 [16–19]. We employed the Cyclin D1 promoter plasmids, −1000/−1 which contains multiple p65 binding motifs cloned in pGL3-Basic vector, to measure promoter activity in response to p65 by a dual luciferase reporter assay. In our study, Fig. 5d, e showed that p65 induced Cyclin D1 promoter transcriptional activity. Co-transfection of the Cyclin D1 promoter with p65 plasmid resulted in ~2.5-fold and ~2-fold induction of promoter activity, respectively. The results again verify that NF-κB-p65 can regulate Cyclin D1 expression at the transcriptional level.

### PAK5 promotes breast cancer growth in vivo

**Fig. 6:**
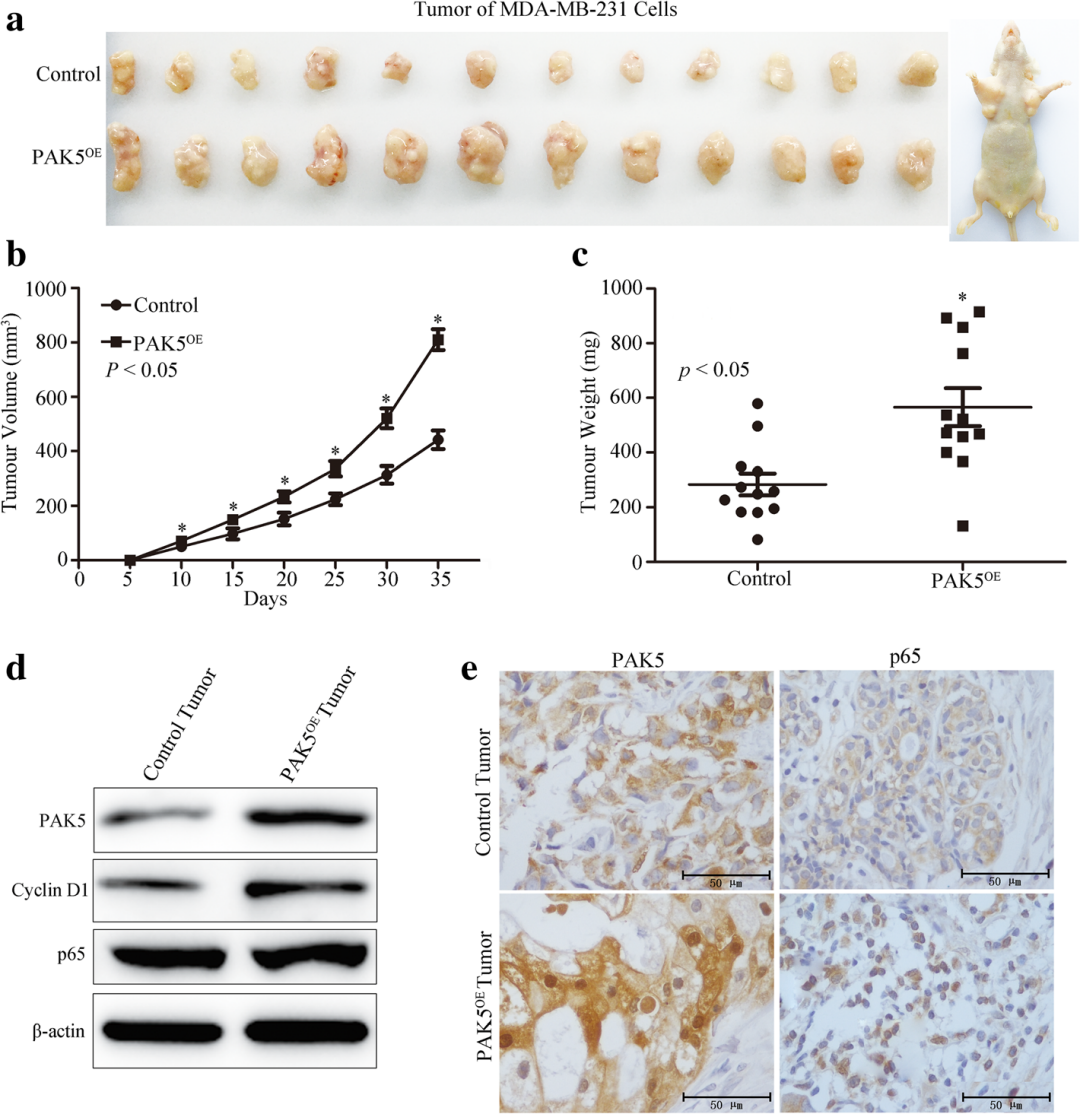
PAK5 promotes the tumor development of mice. **a** Xenograft tumor formation of MDA-MB-231 cells in nude mice. **b, c** The volume and weight of tumor cancer models were analyzed and calculated. **d** Western blotting analyzed the protein levels of PAK5, p65 and Cyclin D1 in harvested tumor tissues. **e** Representative images PAK5 and p65 immunohistochemical staining with PAK5 antibody in control and PAK5^OE^ groups tumor models. *, *P* < 0.05

To further evaluate the role of PAK5 in the breast cancer tumorigenesis in vivo, xenograft cancer models were established by subcutaneously inoculating MDA-MB-231 cells stably transfected with PAK5. In order to minimize the individual variations in animal models, the corresponding control MDA-MB-231 cells transfected with vector plasmid were injected in the left side of the same mice. Compared with control group, the sizes and weights in PAK5 overexpressed xenografted tumors are increased obviously (Fig. 6a-c). The results of Western blot repeatedly indicated that PAK5 enhanced Cyclin D1 level in tumors (Fig. 6d). Subsequently, immunohistochemical staining of xenograft tissues isolated from nude mice showed that with the increase of PAK5 positive signal, p65 positive signal also increased in the nucleus, whereas the p65 signal did not change fundamentally in the cytoplasm (Fig. 6e). These findings were consistent with the in vitro results described above, which firmly validated that PAK5 can activate relocation of the p65 from cytoplasm to the nucleus and then promotes breast cancer cell proliferation.

## Discussion 

An increasing body of evidence suggests that increased expression of PAK5 was observed in a variety of human malignancies including breast cancer, colorectal cancer [20–22], stomach cancer [3, 23], pancreatic cancer, neuroblastoma [21, 24], osteosarcoma [25] and epithelial ovarian cancer [26]. These observations suggested that PAK5 might play a role in tumorigenesis and development. However, the PAK5 expression status and its correlation with the clinicopathologic features in breast cancer have never been illuminated. In the present study, we used TMA technology and immunohistochemistry to investigate the role of PAK5 in breast cancer. Our data demonstrated that PAK5 expression was increased in breast cancer tissues compared with tumor adjacent normal breast tissues. Furthermore, we evaluated the relationship between PAK5 expression and clinicopathologic characteristics. Our results showed that increased PAK5 expression was closely associated with pT status, pN status and TNM stage, which suggest that PAK5 may play an important role in the breast cancer development and progression, as well as may serve as a molecular prognostic marker for this disease. As the last identified member of the PAK family, PAK5 was initially discovered in the brain and promotes neurite outgrowth in nerve cells [3]. The subsequent studies show that PAK5 could protect cells from apoptosis, promoting cell survival by directly phosphorylating BAD on serine 112 and indirectly phosphorylating serine 136 via the Akt pathway, respectively. In addition, PAK5 regulate cytoskeleton remodeling in tumor cell through either stabilizing microtubules by PAK5-MARKK-MARK-tau cascade or facilitating dynamic changes of actin by inducing the formation of filopodia, dissolving stress fibers and focal adhesions. Reports in recent years have shown that PAK5 can promote proliferation by G1 arrest prevention in types of cancers such as hepatocellular carcinoma [27], gastric cancer [23], glioma [28], and osteosarcoma [25]. Our previous studies also suggested that PAK5 contributed to proliferation and cell cycle regulation in breast cancer [7]. Here we aim to confirm the essential roles of PAK5 on cell proliferation after overexpression or knockdown of PAK5 in breast cancer cell BT549 and MDA-MB-231, as well as to provide further insight into the potential mechanism involved.

To explore the mechanism of PAK5 on cell cycle regulation, we examined cell cycles associated regulatory proteins, particularly the key cell cycle regulators of the G1-S transition. Cyclin Ds, as binding and activating partners of CDK4 or CDK6, constitute a key subset of the regulatory components of the cell cycle engine. Cyclin Ds that contain Cyclin D1, Cyclin D2 and Cyclin D3, are closely related G1 cyclins and are of particular importance to the cancer field [29]. In our study, Cyclin D1 and Cyclin D3 were upregulated, while p21 and p27, the inhibitor of Cyclin/CDK complexes, were downregulated after increasing the expression of PAK5 in BT549 and MDA-MB-231 cells. Inverse results were observed after transiently PAK5 knockdown in the two cells. However, the alteration of PAK5 expression has no effect on protein expression of Cyclin A、Cyclin A1、Cyclin B1 and Cyclin B3. These observations suggested that PAK5-induced cell-cycle progression was resulted from PAK5 positively mediating Cyclin D1 and Cyclin D3 as well as negatively controlling p21 and p27. This result is partly similar to that of our previous study: G1 phase arrest induced by PAK5 silence was due to the downregulation of Cyclin D1 and upregulation of p21. It has also been reported that PAK5 might contribute to gain of tumor growth potential, acting by affecting the expression of Cyclin D1 in gastric cancer [23]. However, it remains to be elucidated how PAK5 regulates Cyclin D1 in breast cancer cell proliferation.

Previous studies have demonstrated that activation of PAK5 signaling leads to activation or inhibition of several transcription factors, including GATA1, EGR1, RAF1 and ATF2, which subsequently enhance or suppress diverse biological activities. However, whether the well-known nuclear factor NF-κB that can promote Cyclin D1 expression is involved in PAK5 signaling has not been documented yet. NF-κB is a ubiquitous factor of transcription regulation and plays momentous roles in the occurrence and development of human cancer. In response to different stimuli, cytoplasmic IκB was phosphorylated and degradated, leading to p65 translocation to the nucleus and the promotion of genes transcription involved in cell survival, proliferation, invasion, and inflammation, including Cyclin D1 [9, 16, 30–32]. Hence, we speculate that PAK5 may regulate the expression or transcription of NF-κB, especially its key subunit p65. Consistent with this idea, our present results showed that p65 was phosphorylated via the stimuli of PAK5 and relocated to the nucleus. Then, p65 bound to Cyclin D1 promoter and facilitated breast cancer cell proliferation in vitro as well as in vivo. PAK5 overexpression significantly increased p65 nuclear accumulation and targeted Cyclin D1 DNA sequences to modulate its transcription. Cyclin D1 described above, which is characterized by enhancing transcriptional regulation increased cell cycle by binding and activating partners of CDK4 or CDK6, augmenting cell proliferation. All things considered, PAK5 promoted expression of p65 and, in turn, Cyclin D1, leading to distinct proliferation of the BC cells. Relevant report showed that activated p65, the major transactivation subunit of NF-κB, translocating from cytoplasm to nucleus, directly bound the human Cyclin D1 promoter and stimulated transcription of Cyclin D1 [16, 17, 33]. This implied that in the process of malignant transformation of BC cells, the activated p65 via phosphorylation mediated by PAK5 relocated from cytoplasm to the nucleus, interacted with Cyclin D1 promoter to enhance its expression, accelerating transition from G1 to S phase, which promoted cell proliferation of breast cancer cells. Thus, these results illustrated that PAK5 positively regulated p65 nuclear translocation and that may regulated breast cancer cells proliferation and cell cycle via a process involving NF-κB-p65-dependent regulation of Cyclin D1. However, the relationships of between the co-expression of PAK5/p65 and patients with breast cancer are ambiguous; and the specific PAK5 phosphorylation sites in p65, still remain to be substantiated.

## Conclusions

Taken together, our results showed that PAK5 is expressed at high levels in breast cancer tissue and that increased PAK5 was significantly correlated with breast cancer progression and was a prognostic factor of worse outcome in breast cancer patients. Furthermore, we demonstrated the activated p65 via PAK5-mediated phosphorylation interacted with Cyclin D1 promoter to promote cell proliferation of breast cancer cells. Our results firstly provided the evidences that PAK5 might represent a new therapy to suppress breast cancer uncontrolled proliferation in vitro as well as in vivo. In a word, these findings will hopefully provide directions for identification of novel biomarkers for breast cancer, as well as a potential therapeutic strategy to help restrain the progression of breast cancer.

## Abbreviations

AP: Alkaline Phosphatase
BCT: Breast cancer tissues
CCK-8: Cell Counting Kit-8
co-IP: Co-immunoprecipitation
IRS: Immunoreactive score
IκB: Inhibitor of κB
NF-κB: Nuclear factor-kappaB
NTs: Non-cancerous tissues
PAK5: p21^cdc42/rac1^-activated kinase 5
RHD: Rel homology domain
SD: Standard deviation
Sp: Streptavidin-peroxidase
TMA: Tissue microarray
TNM: Tumor node metastasis
